# Graphene Quantum Dots prepared by Electron Beam Irradiation for Safe Fluorescence Imaging of Tumor

**DOI:** 10.7150/ntno.67070

**Published:** 2022-01-01

**Authors:** Honghong Cao, Wei Qi, Xudong Gao, Qiang Wu, Longlong Tian, Wangsuo Wu

**Affiliations:** 1 Lanzhou University Second Hospital, Lanzhou University, Lanzhou, 730000, China.; 2 Institute of National Nuclear Industry, Lanzhou University, Lanzhou, 730000, China.; 3 Frontiers Science Center for Rare Isotopes, Lanzhou University, Lanzhou, 730000, China.; 4 Lanzhou Resources & Environment Voc-Tech University, Lanzhou, 730000, China.; 5 Hubei Key Laboratory of Bioinorganic Chemistry & Materia Medica, School of Chemistry and Chemical Engineering, Huazhong University of Science and Technology, Wuhan, 430000, China.; 6 CAS Key Laboratory of Chemistry of Northwestern Plant Resources, Institute of Chemical Physics, Chinese Academy of Sciences (CAS), Lanzhou, 730000, China.

**Keywords:** Graphene quantum dots, Red luminescence, Electron beam irradiation, Tumor imaging

## Abstract

Graphene quantum dots (GQD) have attracted much attention due to their unique properties in biomedical application, such as biosensing, imaging, and drug delivering. However, scale preparing red luminescing GQD is still challenging now. Herein, with the help of electron beam irradiation, a simple, rapid, and efficient up-to-down strategy was developed to synthesize GQD with size of 2.75 nm emitting 610 nm luminescence. GQD were further functionalized with polyethylene glycol (PEG) and exhibited good solubility and biocompatibility. The potential *in vivo* toxicity of PEGylated GQD could completely be eliminated by the clinic cholesterol-lowering drug simvastatin. PEGylated GQD could selectively accumulate in tumor after intravenous injection as a security, reliable and sensitive tumor fluorescence imaging agent. Therefore, this work presented a new method preparing red luminescing GQD for biomedical application.

## Introduction

Cancer is a major disease seriously threatening human life and health 1-3. Early diagnosis is an effective method to reduce cancer mortality, improve the survival rate, and prolong life of patient 3, 4. Compared with the traditional diagnosis methods (such as dripping-blood chip detection 5, genetic testing 6, biopsy 7, computed tomography 8, B-ultrasound 9, magnetic resonance imaging 10), fluorescence imaging is a low-cost, high-precision and non-invasive diagnostic technology 11. Quantum dots as a kind of quasi-zero-dimensional nanomaterials break through the limitation of dimensionality, and showing high yield of fluorescence quantum, stable optical properties, and resistance to photobleaching 12. Graphene quantum dots (GQD), as an emerging fluorescence nanomaterial, possess many excellent characteristics such as strong luminous intensity and stability, chemical redox inertness and easy chemical modification 13-17, showing great potential of applications in bioimaging, biosensor and drug deliverer 18, 19.

The existing preparation methods of GQD mainly focuses on the hydrothermal cutting method, oxidative cutting carbon fiber method, and electrochemical peeling method 20-22. The hydrothermal cutting method is similar to the oxidative cutting carbon fiber method, and the method is a classic method for preparing GQD, but the method is relatively cumbersome, and a variety of strong acids are introduced 23. The electrochemical stripping method requires a long time for pre-processing graphite, slow process of post-processing products, and low synthesis yield 24. Therefore, it is still a great challenge for researchers to develop a simple, rapid, scale, and efficient preparation method of GQD.

Radiation synthesis technologies have attracted great attention due to its advantages, such as rapid, efficient and clean industrial potential 25, 26. The electron itself is a very strong reducing agent, so the extra chemical reductant does not need to be added during the synthesis processes 27. Moreover, the morphology and size could be adjusted by controlling the radiation dose, which provides potential method for the preparing small-size GQD 28.

**Figure 1:**
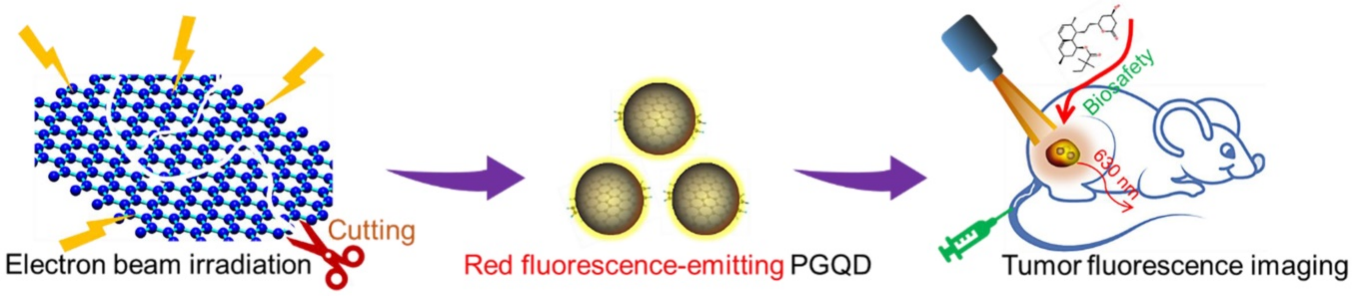
Schematic diagram of irradiation synthesis of graphene quantum dots for tumor fluorescence imaging.

Therefore, in this study, radiation synthesis technology was successfully used to prepare red luminescence GQD with size of 2.75 nm. GQD were further functionalized with polyethylene glycol (PEG) and exhibited good solubility and biocompatibility 29. The potential *in vivo* toxicity of PEGylated GQD (PGQD) could completely be eliminated after injection of clinic cholesterol-lowering drug simvastatin (Supplementary Information Figure S1) 15, 16, 29. PEGylated GQD were found to selectively accumulate in tumor after intravenous injection and could act as a sensitive tumor fluorescence imaging agent (Figure 1). Therefore, this work presented a new method preparing red luminescing GQD for biomedical application.

## Results and Discussion

### Synthesis and characterizations of PGQD

**Figure 2:**
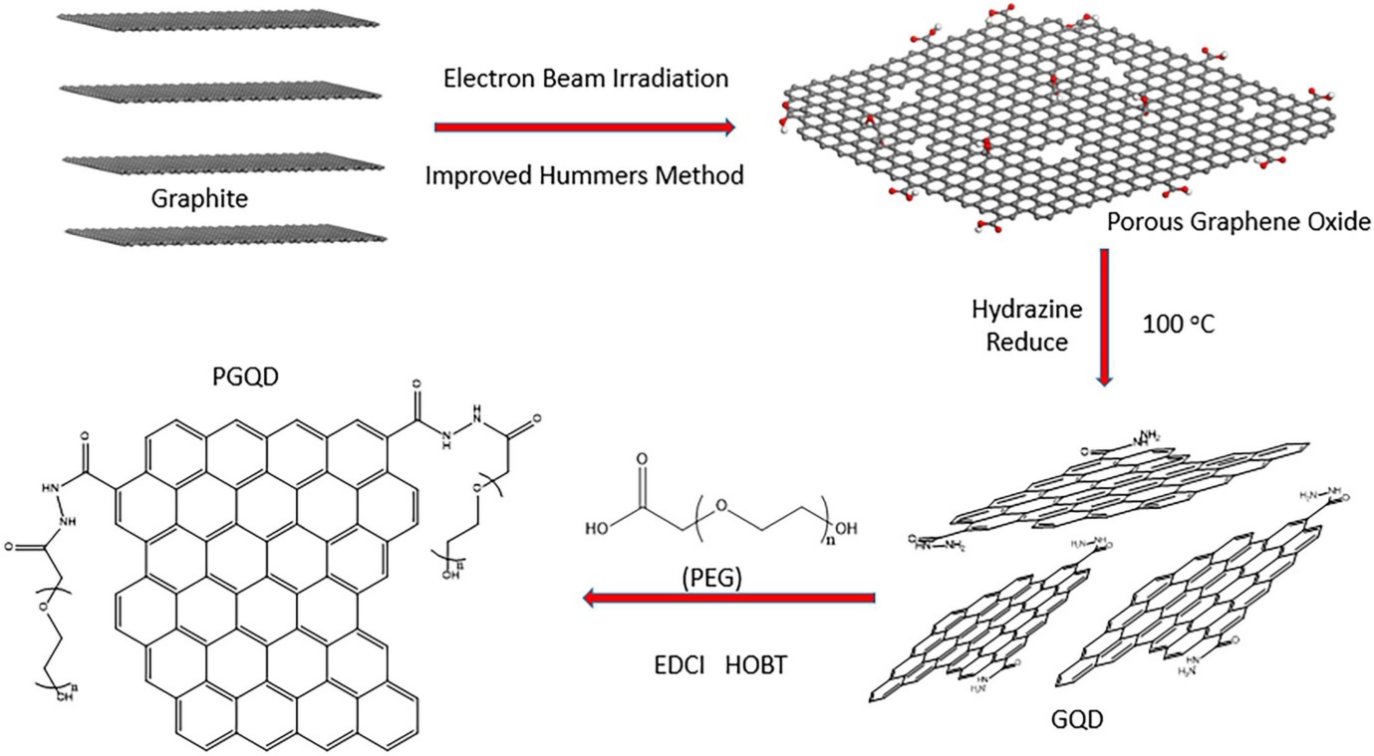
Schematic illustrating the fabrication process of GQD and PGQD.

**Figure 3:**
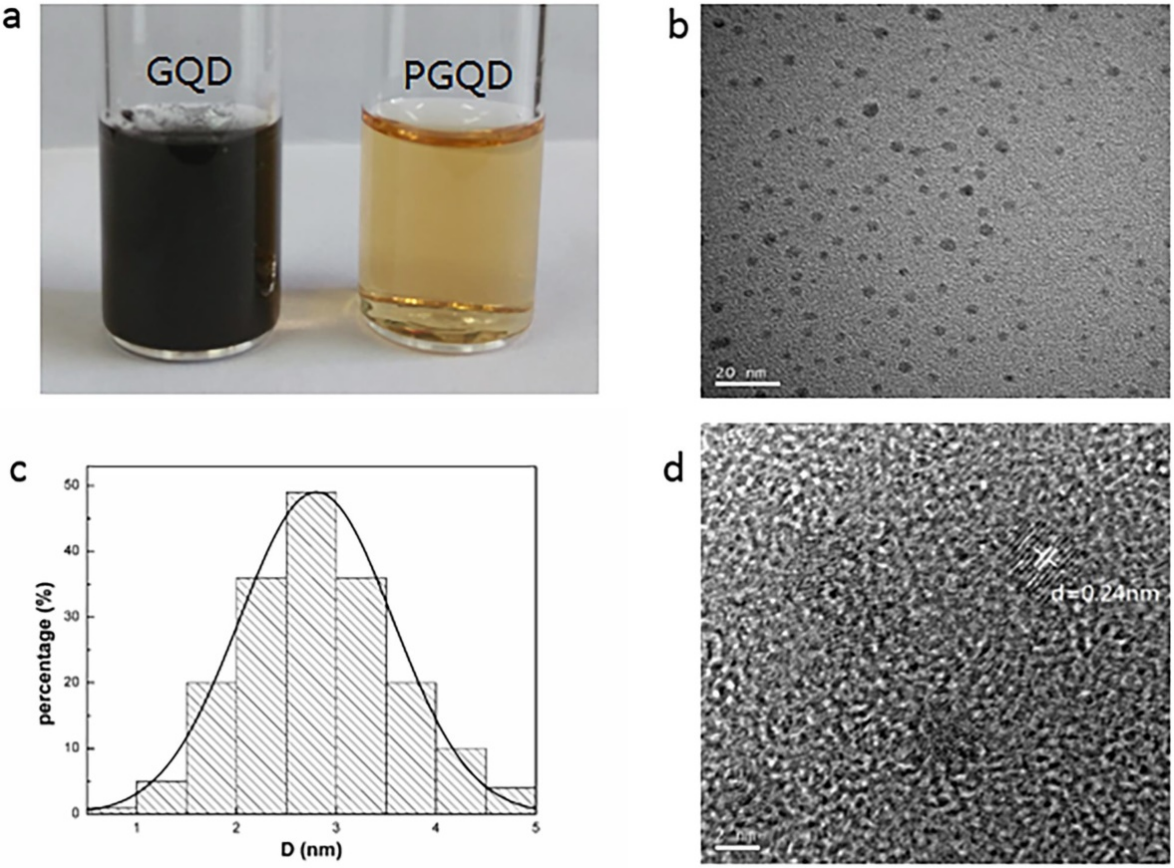
** (a)** 2 g/L GQD and PGQD solution.** (b)** TEM image. **(c)** Size distribution.** (d)** HR-TEM image of PGQD.

**Figure 4:**
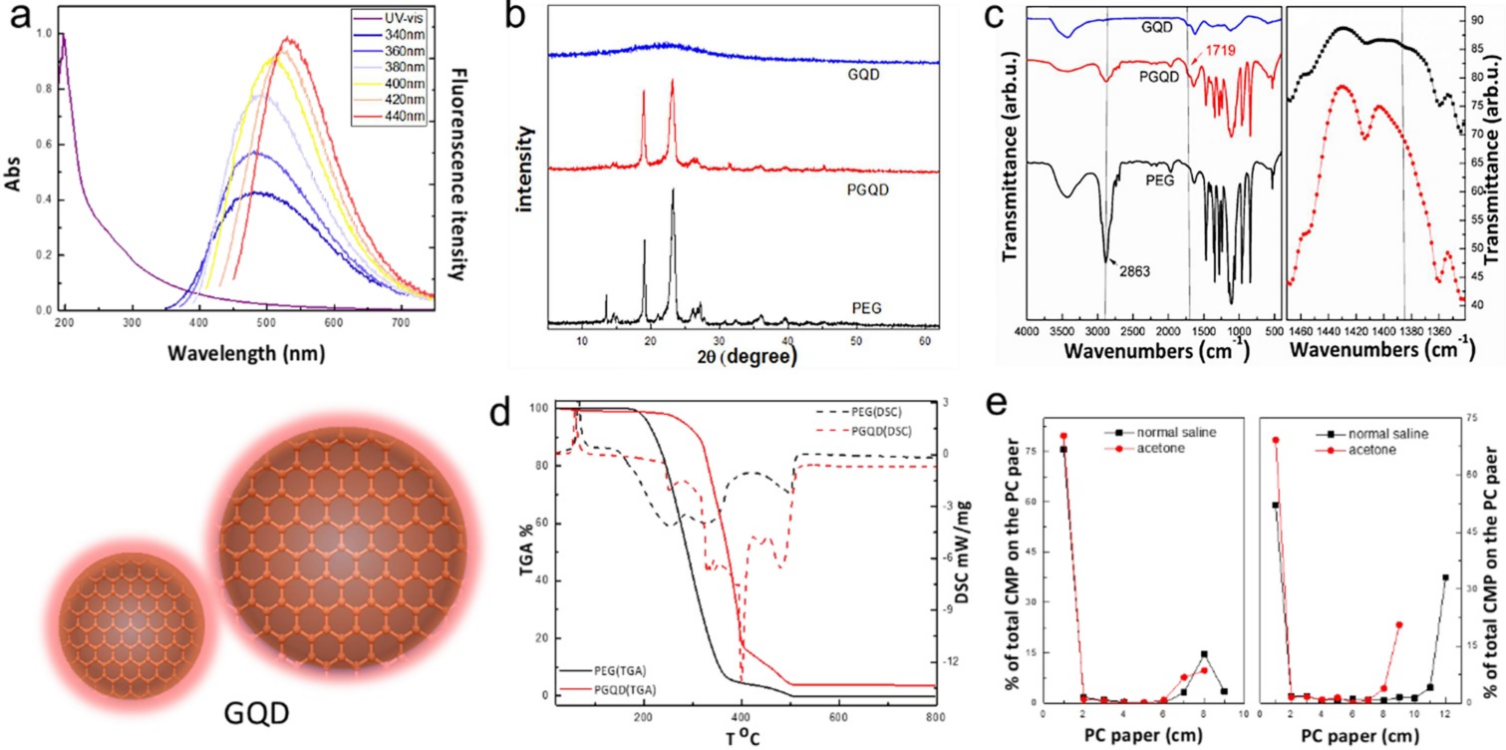
** (a)** UV-vis and PL spectra of PGQD. **(b)** XRD. **(c)** FT-IR of GQD, PGQD and PEG.**(d)** TGA and DSC of PGQD and PEG. **(e)** The determination radiolabling yields of GQD (left) and PGQD (right) by paper chromatography in normal saline and acetone.

In this work, GQD were synthesized by cutting electron beams-irradiated graphite (Figure 2 and Figure S2) 30. GQD were modified with PEG for improving its water solubility and biocompatibility 31, so the color of GQD and PEGylated GQD (PGQD) were black and yellowish brown, as shown in Figure 3a. Transmission electron microscope (TEM) images of PGQD shows a narrow size distribution between 1 and 5 nm with average size diameter of ≈2.75 nm. The high-resolution TEM (HR-TEM) image (Figure 3d) indicates high crystallinity of the PGQD, with a lattice parameter of 0.24 nm, which was the (002) lattice fringes of graphene 32-34. The UV-vis and fluorescence spectra of the PGQD were shown in Figure 4a. An obvious absorption peak could be observed at ≈200 nm, which could be attributed to π-π* transition from benzene structures, and the weak peak at ≈280 nm was from chemically reduced graphene 35, 36. When the excitation wavelength was changed from 320 to 420 nm, fluorescence-emission spectra of PGQD shows the excitation-dependent of photoluminiscence (PL) peak. With the increase of excitation wavelength, the intensity of PL peak increases rapidly, and the peak slowly shifted to long wavelengths, ≈6% quantum yield of the GQD could be obtained, suggesting that the yield was relatively high 37. X-ray diffraction (XRD) of PGQD didn't show the typical broad (002) peak (Figure 4b), which could be attributed to the large amount of PEG coating on the surface of GQD. However, the strong D peak (1386 cm^-1^) and G peak (1599 cm^-1^) could be found in Raman spectra (Figure S3) of PGQD, which confirmed the feature structure 38-40. As compared with the Fourier transform infrared (FTIR) spectrum of GO, the strong and broad absorption peak at ≈3400 cm^-1^ from the O-H stretching vibration could be observed in PGQD and PEG 41. There was a sharp adsorption at 2863 cm^-1^ in PGQD and PEG attributing to C-H; a peak of C=O stretching vibration at ≈1719 cm^-1^ could be seen clearly for GO and PGQD samples; a weak adsorption at ≈1385 cm^-1^ in PGQD indicates the presence of the C-N bonds. Thermogravimetric analysis (TGA) from Figure 4d shows that PEG began to pyrolyze at ≈200 °C, but weight loss of PGQD starts at ≈300 °C. A sharp decalescence peaks could be seen in Differential Scanning Calorimetry (DSC) of PGQD at ≈400 °C, which could be attributed to the thermal decomposition of rest GQD. TGA and DSC results suggests that PEG has been functionalized covalently on the surface of GQD, and every PGQD average linked 20 PEG chains (Figure 4d). The characterization results showed that PGQD exhibits uniform size and excellent fluorescence properties.

### *In vivo* biodistribution of GQD and PGQD

**Figure 5:**
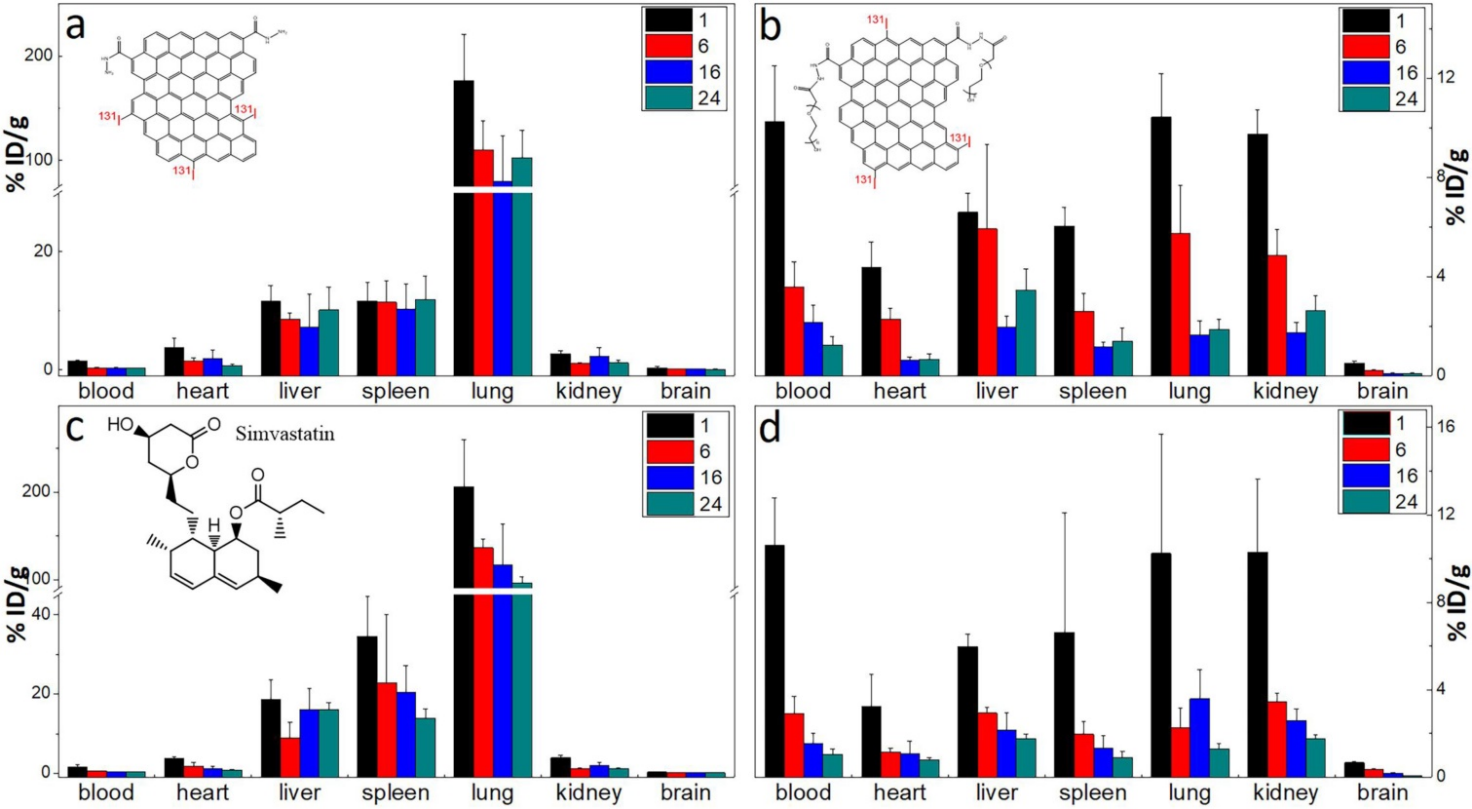
The biodistribution of GQD **(a)** and PGQD **(b)** the effect of simvastatins on the biodistribution of GQD **(c)** and PGQD **(d)** in mice at 1, 6, 16 and 24 h post injection of ^131^I-labeled materials.

The labelling method was used to quantitatively study the biodistribution of GQD and PGQD in mice 42. GQD and PGQD were labeled with ^131^I by chloramine T oxidation method. The radiolabeling yields and stability of GQD were determined by paper chromatography, with saline and acetone as mobile phase (Figure 4e). The radiolabeling yields were over 60%. The distributions (Figure 5a-b) showed that GQD with low solubility were quickly captured by pulmonary capillary bed and mainly accumulated in lung. However, PGQD were found to accumulated in reticuloendothelial system and kidney, suggesting that PGQD with small size could also be excreted through the kidney. The distribution of GQD and PGQD were independent with simvastatin injection (Figure 5c-d), but the clearance rates of GQD and PGQD were obviously improved after simvastatin injection, especially in lung and spleen, which might reduce the *in vivo* toxicity of GQD and PGQD.

### Serum biochemistry

**Figure 6:**
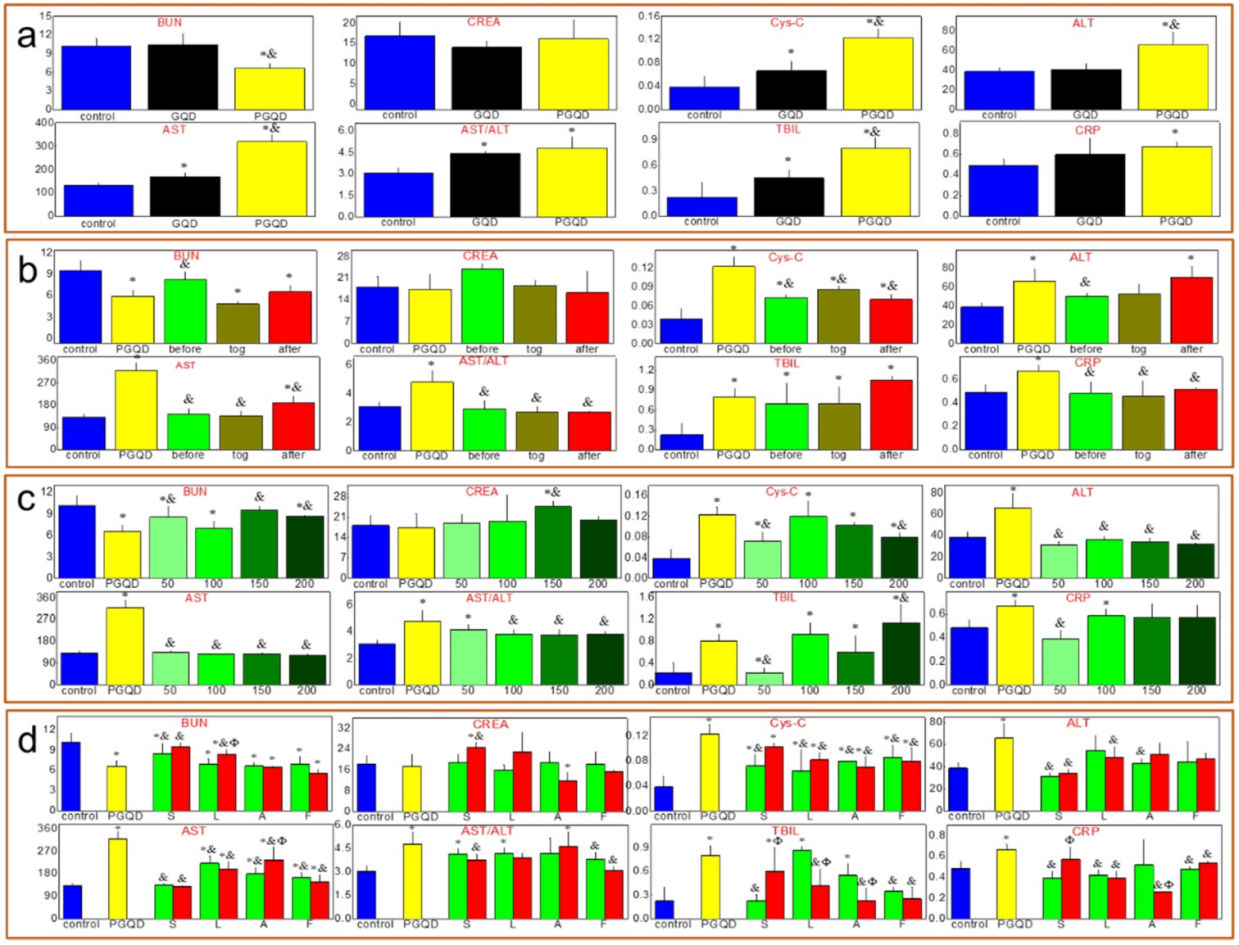
** (a)** Serum biochemistry test of mice injected normal saline (control group), GQD or PGQD. ^*^p<0.05 compared with the control groups. ^&^*p*<0.05 compared with GQD group. n=5-6. **(b)** The effect of injection schedule of simvastatin and PGQD on toxicity.^ *^p<0.05 compared with the control groups. ^&^*p*<0.05 compared with PGQD group. n=5-6. **(c)** Effect of dose of simvastatin on the toxicity of PGQD.^ *^p<0.05 compared with the control groups. ^&^*p*<0.05 compared with PGQD group. n=5-6. **(d)** Effect of four statins (dose of 50 and 150 µg) on the toxicity of PGQD.^ *^p<0.05 compared with the control groups. ^&^*p*<0.05 compared with GQD group. ^Φ^*p*<0.05 compared the dose of 50 µg statins groups. n=5-6.

Serum biochemistry test (Figure 6a) was conducted to study the potential toxicity of GQD and PGQD in mice *in vivo*. As a sensitive indicator of kidney functions, the Cystatin C (Cys-C) in the blood increased. Some liver function markers were also tested, such as alanine aminotransferase (ALT), aspartate aminotransferase (AST) and total bilirubin (TBIL). The increase of AST and TBIL indicated hepatic toxicity after GQD and PGQD injection. Therefore, statins were used to eliminate the accumulation and toxicity of GQD and PGQD 41, so the injection sequence of simvastatin and nanoparticles was first investigated (Figure 6b) 43. Simvastatin (150 μg, 10 mg/kg) was injected at 5 h before, together with, and 5 h after the injection of GQDP/GQD (400 μg, 25 mg/kg), then mice were sacrificed at 24 h for collecting blood (5 mice per group). Cys-C, AST and TBIL in the blood of mice treated by simvastatin and GQD together decreases significantly, indicating that simvastatin could improve the function of kidney and liver, and Cys-C, ALT, AST and C-reactive protein (CRP) in the blood of PGQD group mice decreases significantly after treatment of simvastatin. The results indicate that combined injection of simvastatin and GQD/PGQD could significantly alleviate the toxicity of GQD/PGQD* in vivo*.

The effect of simvastatin dose on toxicity *in vivo* was also investigated as shown in Figure 6c. Blood urea nitrogen (BUN), serum creatinine (CREA), ALT and AST of GQD increases unexpectedly by injection of simvastatin with 200 μg dose, which may be attributed to the drug toxicity of simvastatin. However, importantly, almost all biochemistry parameters have reached normal level for mice combined injected by PGQD with simvastatin, indicating that the toxicity of PGQD could be reduced efficiently by low dose of simvastatin. In general, 50 or 150 μg simvastatin could reduce the toxicity of GQD/PGQD *in vivo*. Moreover, simvastatin, lovastatin, atorvastatin, fluvastatin were chosen to study the effect of statins types on the toxicity of GQD/PGQD *in vivo* (Figure 6d). Mice were injected by GQD/PGQD mixed with 50 or 150 μg statins, then sacrificed at 24 h for collecting blood. Results show that all four statins all could be used to reduce the toxicity of GQD/PGQD *in vivo*.

### Histology Examination

**Figure 7:**
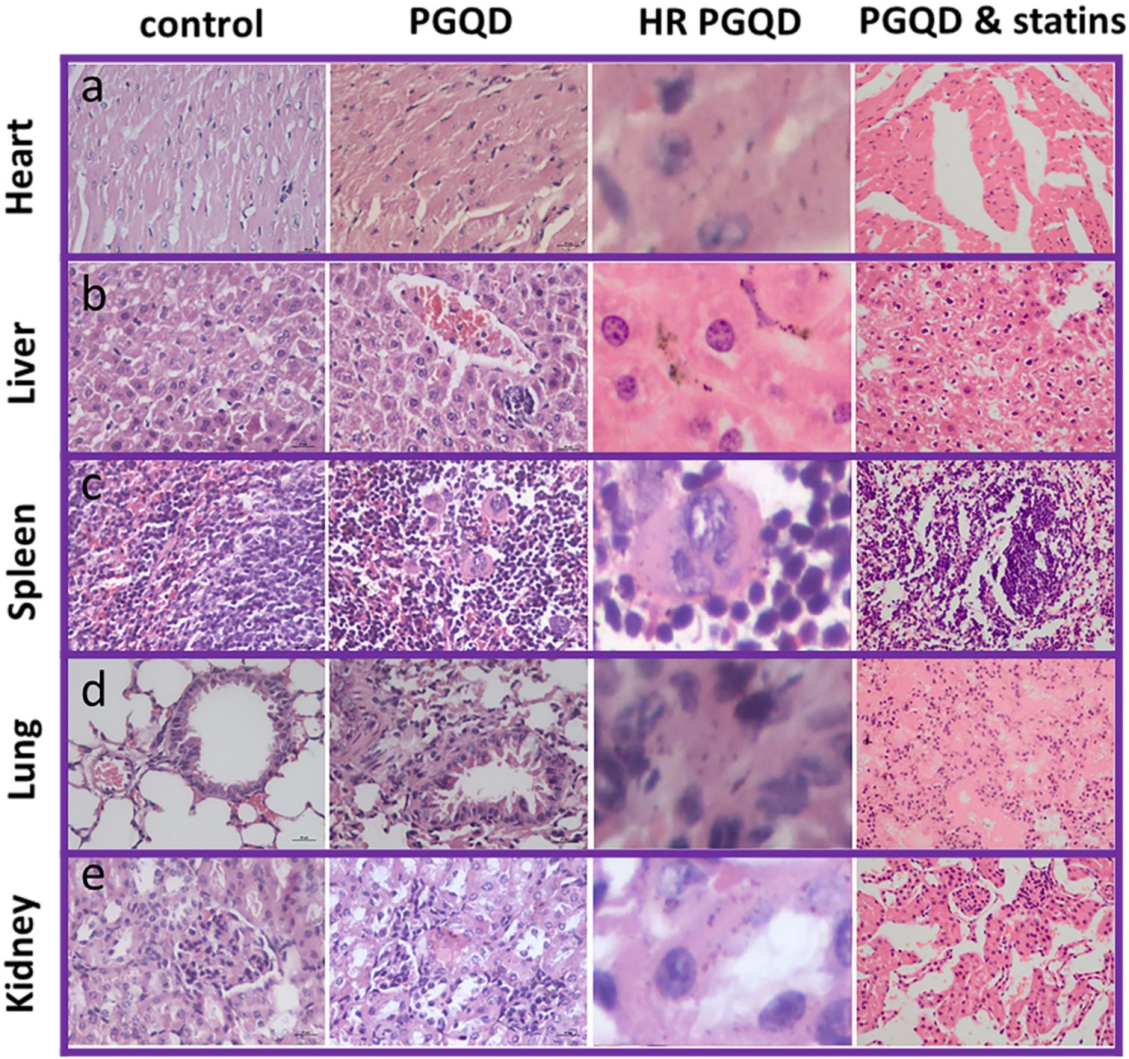
Photographs and high-resolution (HR) photographs of H&E stained tissue slices (heart, liver, spleen, lung and kidney) of mice injected with of PGQD or PGQD mixed with simvastatin.

To further confirm effect of statins on the toxicity of GQD/PGQD *in vivo*, the mice tissues were collected at 24 h after injecting with normal saline, GQD, PGQD, GQD with simvastatin, and PGQD with simvastatin for make slices of Haematoxylin and Eosin (H&E) staining (Figure 7). Large numbers of nanoparticles were observed in the heart, liver, spleen, lung, and kidney tissues of GQD and PGQD group. After simvastatin injection, nanoparticles were eliminated from heart, liver and kidney and nanoparticles-induced damages could be repaired. Meanwhile, nanoparticles were observed much higher in lung than other tissues, which efficiently reduced after co-administration of simvastatin. Histology examination indicates that the nanoparticles accumulated in tissues could be efficiently cleared and tissue damage could be repaired after statins injection.

### Red blood cell (RBC) test

The effect of nanoparticles on RBC has been investigated (Figure S4-5). Optical microscope and SEM images show that the morphology of RBCs remain unchanged after incubation with 100 μg/mL of GQD/PGQD for 4 h as compared with the control group, which indicates that toxicity of GQD and PGQD to RBCs was not significant.

### Fluorescence imaging of tumor

**Figure 8:**
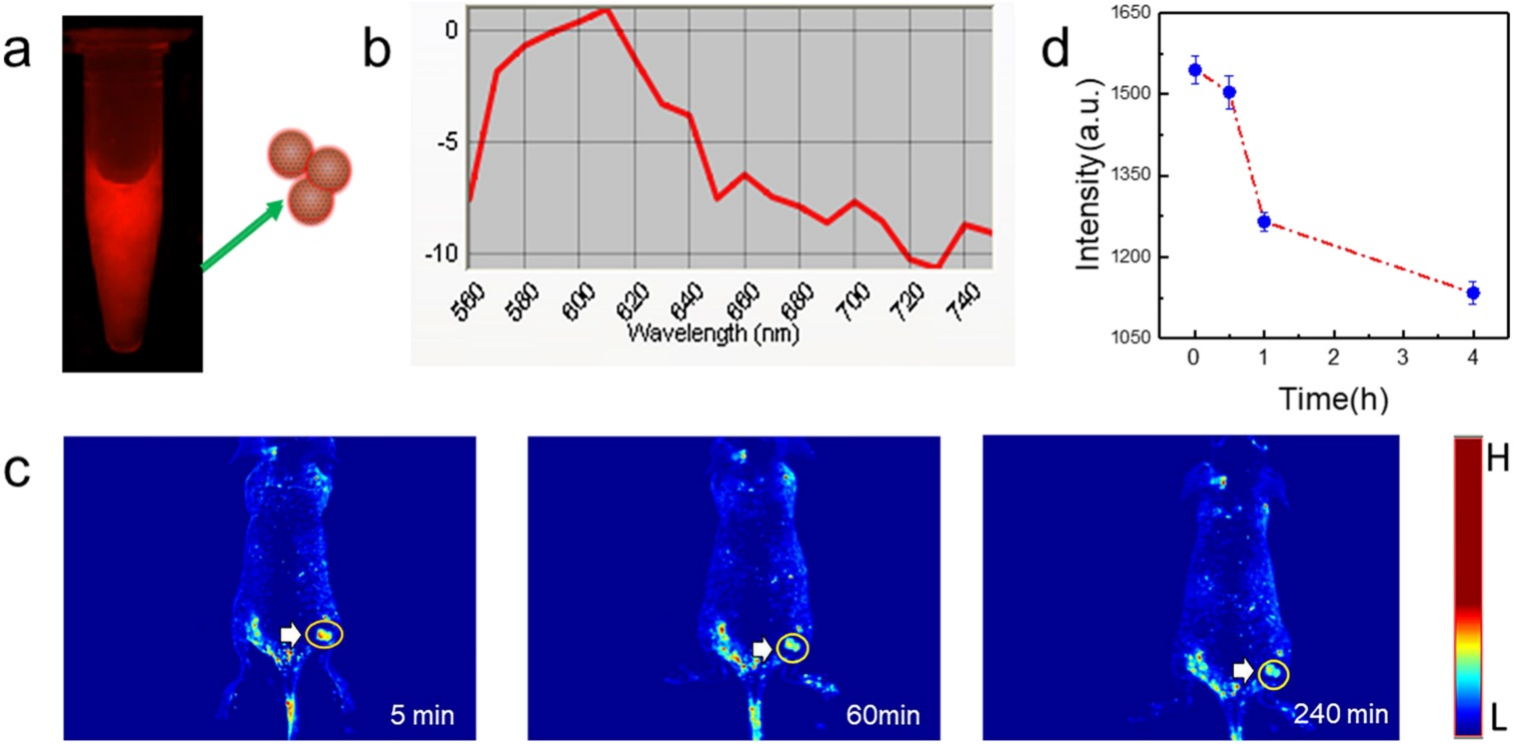
***In vivo* tumor fluorescence imaging with PGQD. (a)** Fluorescence images of PGQD. **(b)** Fluorescence emission spectra of PGQD. **(c)** Mice bearing 4T1 tumors were intravenously injected with PGQD suspension solution and imaged with a small animal fluorescence imaging system at different time points (5 min, 1 h and 4 h). **(d)** Fluorescence signals intensity in tumor.

PGQD could emit strong red fluorescence at about 610 nm (Figure 8a-b). Mice bearing 4T1 tumors were intravenously injected with PGQD suspension solution and imaged with a small animal fluorescence imaging system. After 5 minutes post injection, strong red fluorescence signals were observed in tumor, indicating that PGQD would selectively and rapidly accumulate in the tumor area (Figure 8c). The fluorescence signals in tumor (Figure 8d) were found to decrease with time, indicating that small size PGQD could be gradually eliminated. Those results proved that PGQD prepared by electron beam irradiation could was a good tumor-targeting imaging agent.

## Conclusion

Graphene quantum dots were successfully prepared with electron beam irradiated graphite. The obtained GQD exhibited uniform size and excellent fluorescent property. GQD were further functionalized with polyethylene glycol (PEG) and exhibited good solubility and biocompatibility. The potential *in vivo* toxicity of PEGylated GQD (PGQD) could be completely eliminated after injection of clinic cholesterol-lowering drug simvastatin. PEGylated GQD were found to selectively accumulate in tumor after intravenous injection and could act as a sensitive tumor fluorescence imaging agent. This work presented a new method preparing red luminescing GQD for biomedical application.

## Materials and methods

### Synthesis and characterization of GQD and PGQD

In our previous work 44, heavily oxidized graphene oxide (HGO) with lots defects was prepared by the improved Hummers method with electron beam irradiated graphite. A 0.4 MeV linear electron accelerator (USA, WASIK, P=16 KW) was used here. Before the GQDs preparation, the graphite flake (about 5 g) was packaged by a 10×10 cm PE bag and irradiated at 5 MGy with a dose rate of 8 kGy. GQD was obtained through directly reducing HGO by hydrazine at 100 °C for 24 h. 200 mg of carboxylated PEG (3350 Da) was activated with 20 mg of 1-Ethyl-3-(3-dimethyllaminopropyl) carbodiie hydrochlide (EDCI) in 50 mM phosphate buffer (pH 5.5). After 15 min, 20 mg of GQD was added into the solution and further stirred for 2 h for complete reaction. At last, the solution was concentrated and rinsed with water through spin-dialysis (MWCD=8000-14000 Da) to obtain PGQD 45.

The prepared GQD and PGQD was collected and characterized by UV-Vis spectra, Fluorescence spectra, Transmission electron microscopy (TEM), X-ray Diffraction (XRD), Raman spectroscopy, Fourier transform infrared (FTIR) spectroscopy. UV-Vis spectra were collected with a PerkinElmer Lambda 35 spectrophotometer. Fluorescence emission spectra were recorded with a FLSP 920 fluorescence spectrometer (Edinburgh Instruments Ltd.). TEM was examined using a Tecnai-G2-F30Field Emission Transmission Electron Microscope (FEI Corporation). XRD was scanned from 5° to 60° on powder X-ray diffractometer (Panalytical). Raman spectra from 500 to 4000 cm^-1^ were collected on an in Via-Reflex Raman scope using a 632.8 nm He-Ne laser (Renishaw). FTIR spectra were recorded from 400 to 4000 cm^-1^ on a NEXUS 670 5-DX 170SX spectrometer (Nicolet Instrument Corporation).

### ^131^I Labeling of GQD and PGQD

The GQD (2 mg/mL) was labeled by ^131^I using a standard chloramine T oxidation method 42, 46, 47. A mixture of 5mL of GQD (2 mg/mL), 1 mCi Na^131^I and 100 μL 4 mg/mL chloramine-T (Sigma-Aldrich) was reacted in a pH 7.5 phosphate buffer (0.02 M) for 30 min at room temperature. Excess ^131^I was completely removed by centrifugation with water for 4~6 times until no detachable gamma activity in the filtrate solution. The PGQD was labeled by the same method, while the excess ^131^I was completely removed with ultrafiltration centrifuge tube (MWCO=3 kDa) for 24h. Radiolabeling stability was tested with paper chromatography (saline and acetone as mobile phase).

### Biodistribution of ^131^I-Labeled GQD and PGQD and the influence of simvastatin

Kunming mice initially weighing 15 g to 18 g were provided by the Laboratory Centre for Medical Science, Lanzhou University, Gansu, China. All animals were housed in individual cages in a temperature-controlled (21 °C to 22 °C) and light-controlled (turned on from 08:00 h to 20:00 h) environment and were fed food and tap water ad libitum. All animal protocols were in accordance with the European Communities Council Directive of November 24, 1986 (86/609/EEC) and approved by Institutional Animal Care and Use Committees of Gansu Province Medical Animal Center and Lanzhou University Animal Committees Guideline (China). The body weights of the mice were determined before they were injected intravenously (i.v.) with 20 mg/kg.bw of ^131^I-Labeled GQD or PGQD solution (injection volume of 0.2 ml, GQD and PGQD groups were diluted from 2 mg/mL to 0.8 mg/mL by saline, while the simvastatin groups were diluted from 2 mg/mL to 0.8 mg/mL by 0.5 mg/mL simvastatin). Mice were sacrificed at 1, 6, 16 and 24 h after injection. Tissues from the heart, lungs, liver, spleen, kidney and brain were immediately dissected. Each tissue or organ was wrapped in foil, weighed and counted. Data were corrected for physical decay of radioactivity. The distribution in the tissues was presented in percent injected dose per gram of wet tissue (%ID/g), which could be calculated by the percent injected dose (tissue activity/total activity dose) per gram of the wet tissue 48. Statistics were based on standard deviations of 4~6 mice per group.

### Blood biochemistry analysis and Histology Examinations

Healthy female Kunming mice were injected with 500 uL of 0.8 mg/mL GQD or PGQD (a dose of 20 mg/kg) and statins was injected under different doses or sequence (before, together or after injecting GQD or PGQD). Mice were sacrificed at 24 h after injection. Other five healthy Kunming mice were used as the untreated control and sacrificed at 24 h. An approximate 0.5 mL portion of blood from each mouse was collected for a blood biochemistry analysis before the mouse was euthanatized. The serum biochemistry data was measured in the Lanzhou University Second Hospital. Major organs (including liver, kidneys, spleen, heart and lung) from those mice were harvested and fixed in formalin for hematoxylin and eosin (H&E) staining. Statistics were based on standard deviations of 5 mice per group.

### RBC test

Fresh blood was obtained from Kunming mice, anticoagulated with heparin and centrifuged at 4000 rpm for 5 min. The plasma was removed. The separated erythrocytes were washed three times by centrifugation (4000 rpm, 5 min) in 10 volumes of PBS. The supernatant and buffy coat of white cells were carefully removed with each washing. Washed erythrocytes were finally re-suspended with the same buffer and stored at 4 °C and used within 6 h. The erythrocyte suspension was incubated with 100 μg/mL of GQD, PGQD. At 4 h, 100 μL of these mixtures were dropped onto a glass slide and observed by optical microscopy and confocal laser scanning microscope. The incubated suspensions were fixed with a glutaraldehyde solution (3%), followed by centrifugation (4000 rpm, 5 min), and then washed with gradient ethanol. Afterwards, the samples were vacuum-dried for SEM characterization 49.

### Fluorescence Imaging of Tumor

Female nude mice were purchased from Nanjing Peng Sheng Biological Technology Co. Ltd. and used under protocols approved by Lanzhou University Laboratory Animal Center. 1 × 10^6^ 4T1 cells were suspended in 50 μL PBS, and then injected subcutaneously into the back of each mouse. When the tumor volume reached about 100 mm^3^, the mouse was intravenously injected with PGQD and imaged with Maestro™ In-Vivo Imaging System at different time points post injection.

### Statistical Analysis

The experimental data is sorted by excel and reported data as mean values ± SD of multiple determinations. All statistical analysis were carried out using SPSS software. Through the Kplmogorov-Smirnov Npar test and the homogeneity of variance test, the statistical significance in the difference was evaluated by analysis of variance (ANOVA) with the significance level of 0.05 or Kruskal-Wails-test method.

## Supplementary Material

Supplementary figures.
