# Supplementary material for: Graphene Quantum Dots prepared by Electron Beam Irradiation for Safe Fluorescence Imaging of Tumor
Source: Nanotheranostics. 2022 Jan 1;6(2):205–14. doi: 10.7150/ntno.67070 (PMC8671948; doi:10.7150/ntno.67070)
Supplement: Supplementary file 1 — Supplementary figures. [file ntnov06p0205s1.pdf]

Supplement information

S1, structure of statins

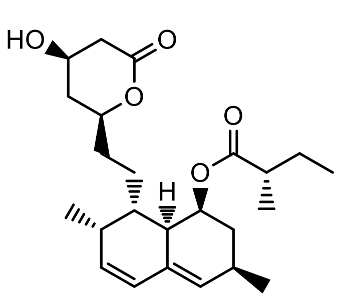

Simvastatin

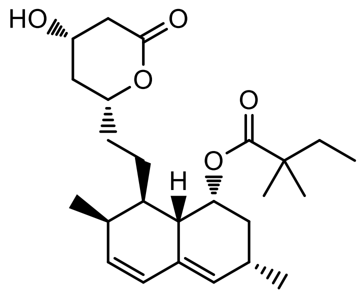

Lovastatin

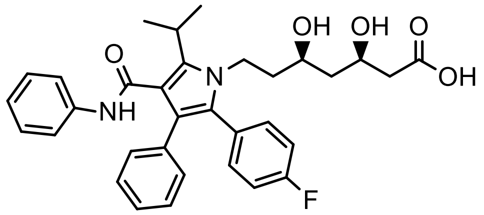

Atorvastatin

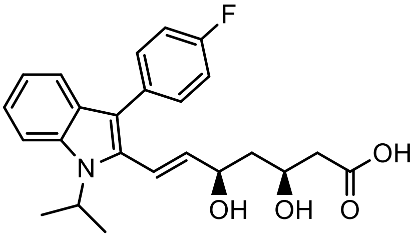

Fluvastatin

Figure S1. Chemical structure of simvastatin, lovastatin, atorvastatin and fluvastatin

S2, heavily oxidized graphene oxide (HGO) with lots defects

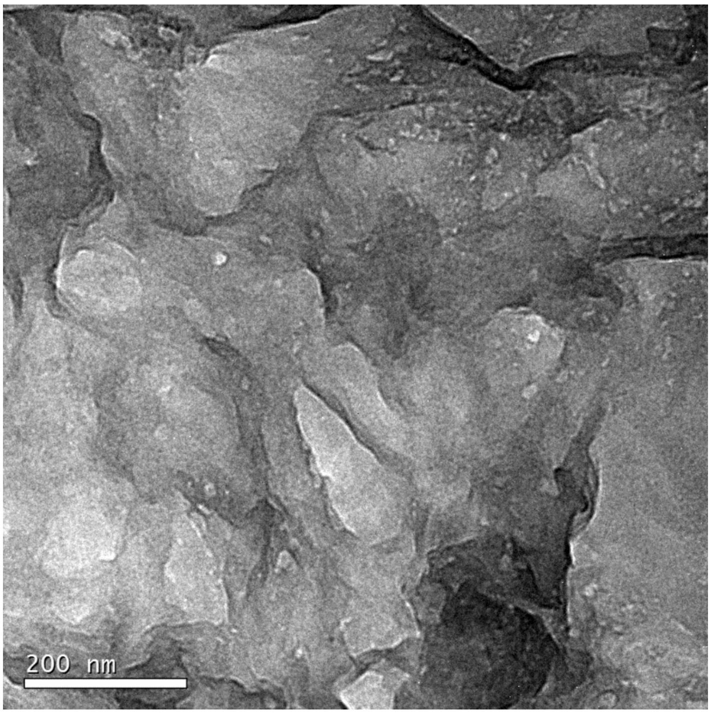

Figure S2. TEM image of heavily oxidized graphene oxide with lots defects

S3, Raman spectra of PGQD and GQD

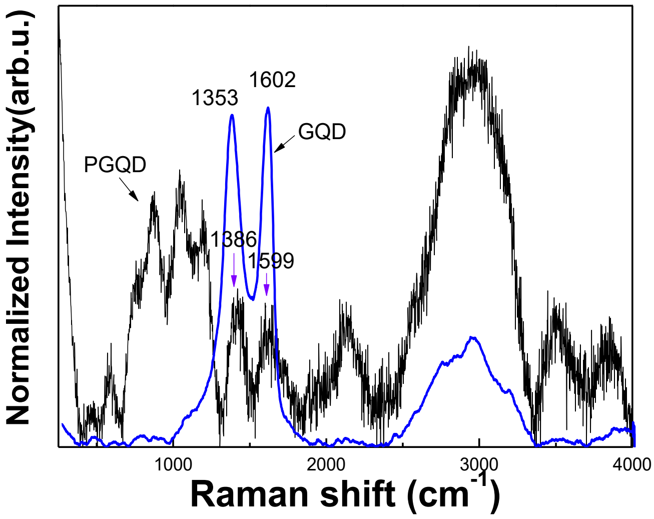

Figure S3. Raman spectra of PGQD and GQD

S4, average PEG chains on PGQD

$$\frac{n \times M_{PEG}}{M_C \times \frac{S_{PGQD}}{S_C}} = \frac{m_{PEG}}{m_{GQD}}$$

Where n represent the average PEG chains linked on PGQD, M_{PEG} represent the molecular weight of PEG (3350), M_C is the atomic weight of carbon (12), S_{PGQD} represent the average square of PGQD calculated by $\pi \times (D)^2/4$, D represent the average diameter of PGQD (2.75 nm), S_C represent single C atom square on graphene calculated by $3 \times \sqrt{3} \times b^2/4$, b represent the C-C bond length (0.14 nm), m_{PEG} represent the weight percentage of PEG in the TGA, m_{GQD} represent the weight percentage of GQD in the TGA.

S5, optical microscope images of RBCs

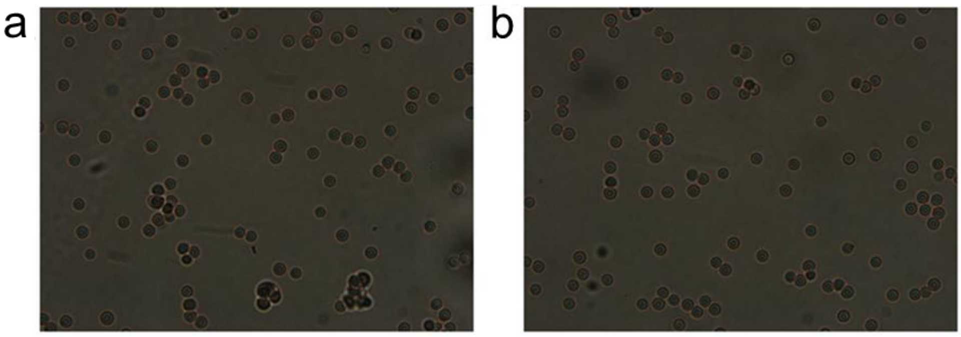

Figure S4. Optical microscope images of RBCs after incubating with GQD. (a) and PGQD. (b) for 4 h.

S6, SEM images of RBCs

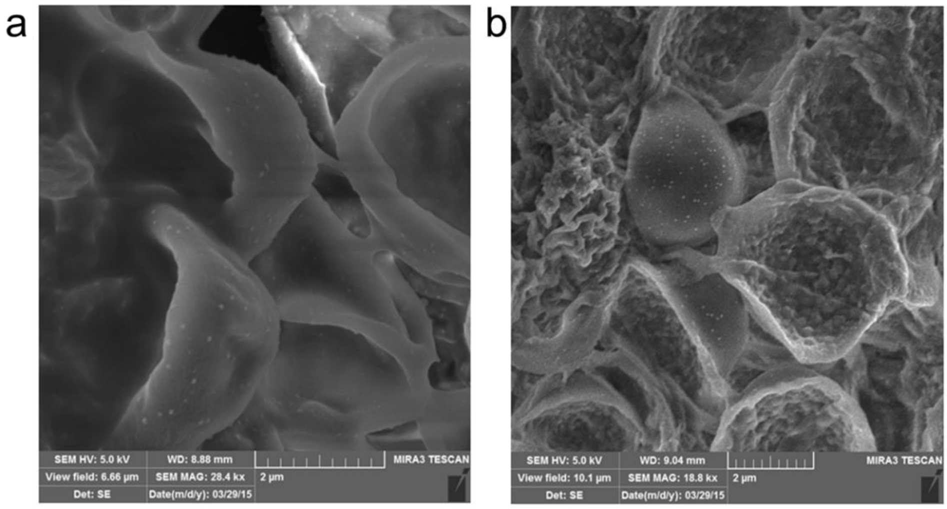

Figure S5. SEM images of RBCs of RBCs after incubating with GQD. (a) and PGQD. (b) for 4 h.

S7, mice were injected five times with GQD

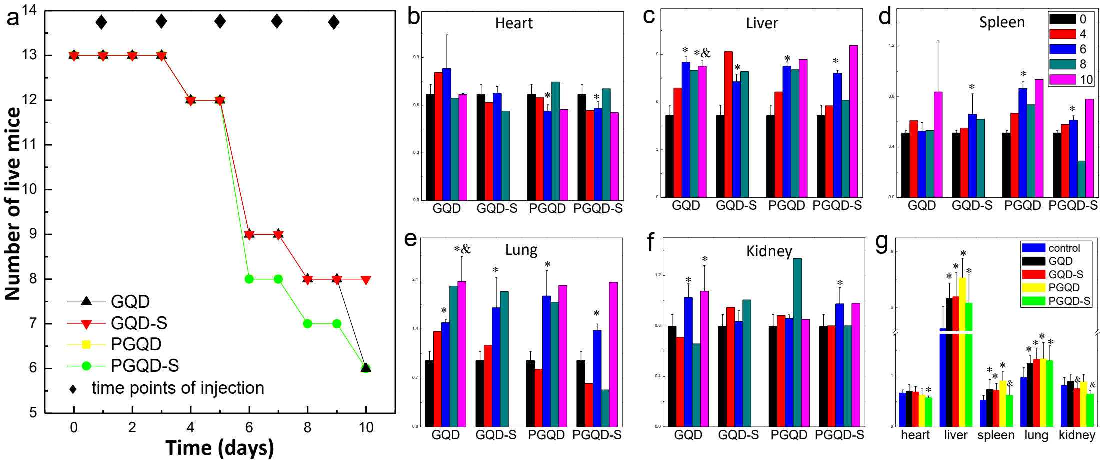

Figure S6. (a)Mice were injected five times with GQD, GQD mixed with simvastatin, PGQD and PGQD mixed with simvastatin. (b-f) The ratio of organs to total weight of the dead mice after injection (4, 6, 8 and 10 day) compared that of mice before injection (0 day). (g) The ratio of organ to total weight of the left half mice after multiple-dose.

S8, the effect of injection schedule of simvastatin and GQD

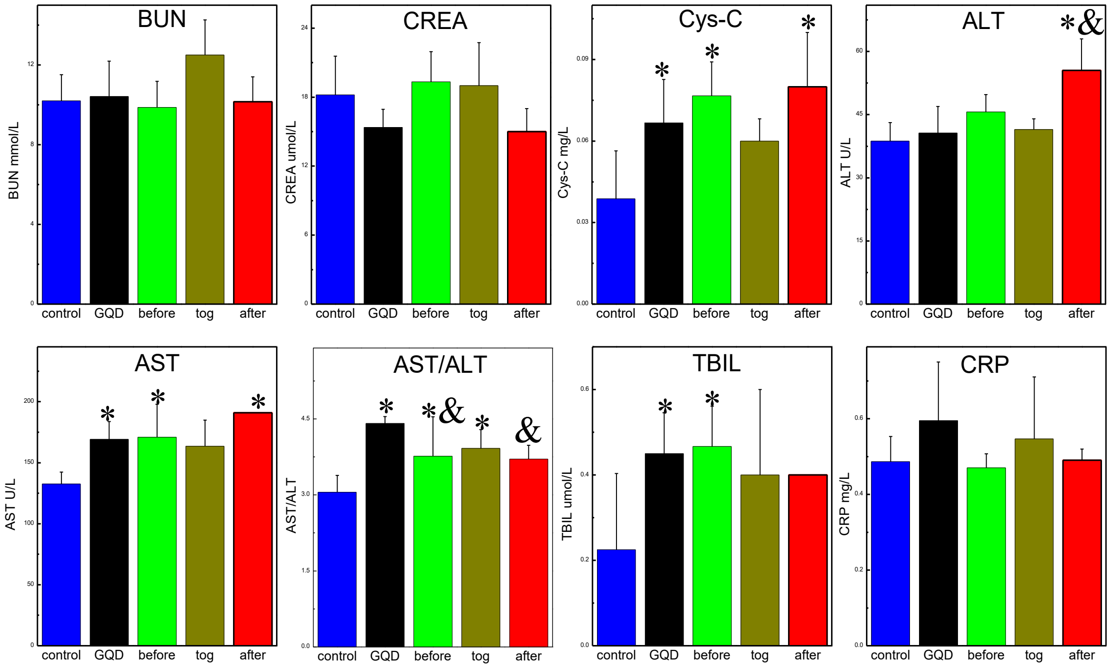

Figure S7. The effect of injection schedule of simvastatin and GQD on toxicity. *p<0.05 compared with the control groups. &p<0.05 compared with GQD group. n=5–6, all data were statistical analysis by ANOVA.

S9, effect of dose of simvastatin on the toxicity of GQD

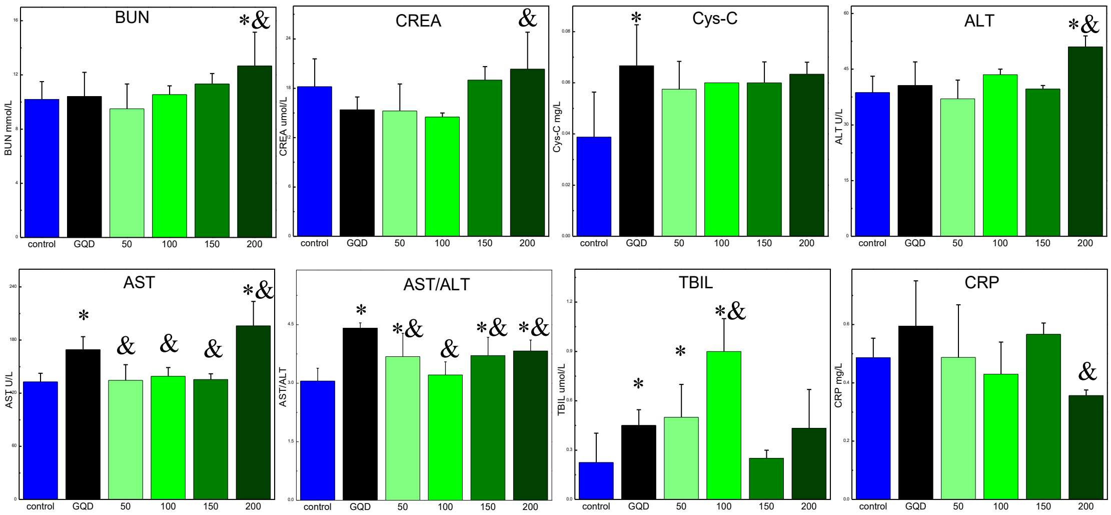

Figure S8. Effect of dose of simvastatin on the toxicity of GQD. *p<0.05 compared with the control groups. &p<0.05 compared with GQD group. n=5–6, all data were statistical analysis by ANOVA.

S10

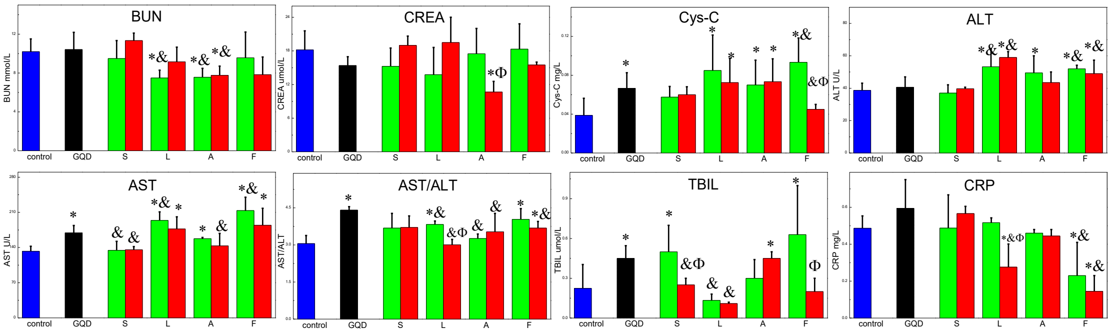

Figure S9. Effect of four statins (dose of 50 and 150 μg) on the toxicity of GQD. *p<0.05 compared with the control groups. &p<0.05 compared with GQD group. Φp<0.05 compared the dose of 50 μg statins groups. n=5–6, all data were statistical analysis by ANOVA.

S11, H&E stained tissue slices

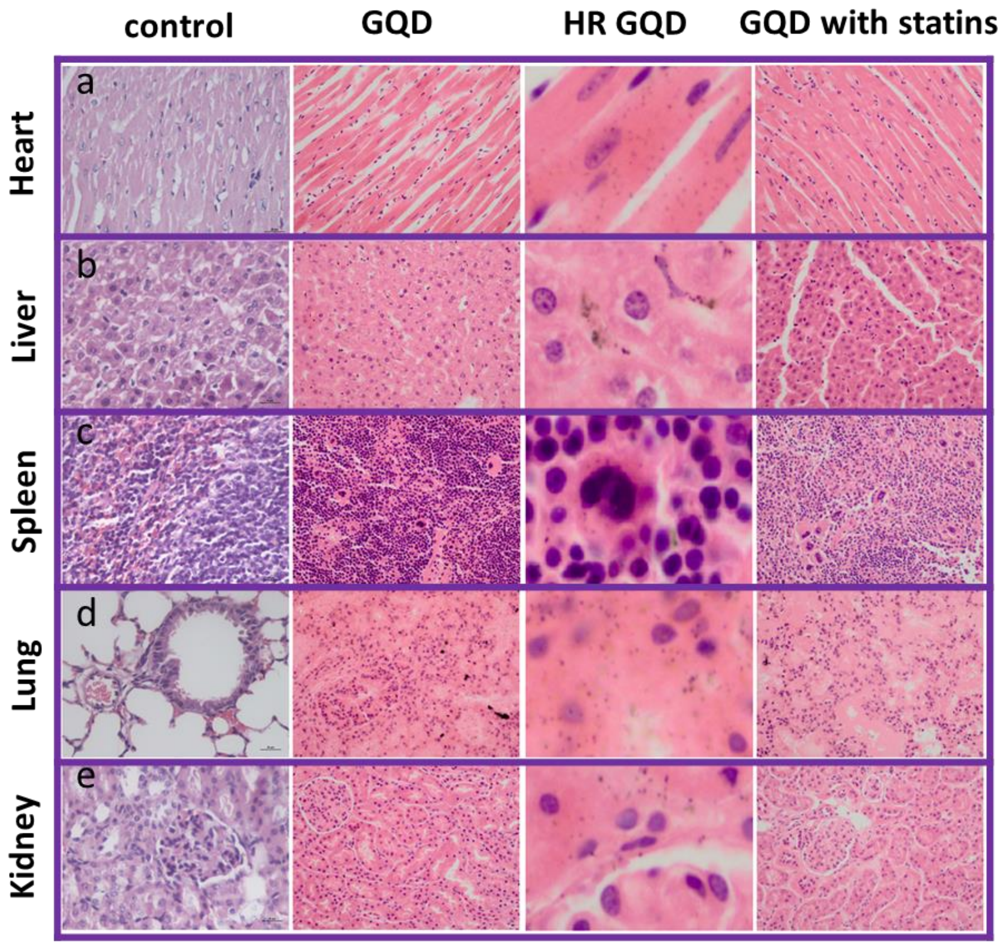

Figure S10. H&E stained tissue slices (heart, liver, spleen, lung and kidney) of mice injected with of GQD or GQD mixed with simvastatin
